# Retinal Microvascular Profile of Patients with Coronary Artery Disease

**DOI:** 10.3390/medicina61050834

**Published:** 2025-04-30

**Authors:** Alexandra Cristina Rusu, Raluca Ozana Chistol, Grigore Tinica, Cristina Furnica, Simona Irina Damian, Sofia Mihaela David, Klara Brînzaniuc, Karin Ursula Horvath

**Affiliations:** 1 Doctoral School of Medicine and Pharmacy, Faculty of Medicine, University of Medicine, Pharmacy, Science and Technology of Targu Mures, 540142 Targu Mures, Romania; alexandracristina.rusu@gmail.com; 2 Faculty of Medicine, “Grigore T. Popa” University of Medicine and Pharmacy, 700115 Iasi, Romania; grigore.tinica@umfiasi.ro (G.T.); cristina.furnica@umfiasi.ro (C.F.); simona.damian@umfiasi.ro (S.I.D.); 3 “Prof. Dr. George I.M. Georgescu” Cardiovascular Diseases Institute, 700503 Iasi, Romania; 4 Institute of Forensic Medicine, 700455 Iasi, Romania; 5 Faculty of Medicine, University of Medicine, Pharmacy, Science, and Technology of Targu Mures, 540142 Targu Mures, Romania; klara.brinzaniuc@umfst.ro (K.B.); karin.horvath@umfst.ro (K.U.H.); 6 County Emergency Clinical Hospital of Targu Mures, 540136 Targu Mures, Romania

**Keywords:** coronary artery disease, cardiovascular diseases, central retinal arteriolar equivalent, central retinal venular equivalent, arteriovenous ratio, fractal dimension, tortuosity, lacunarity

## Abstract

*Background and Objectives*: Screening, primary prevention, and the early identification of high-risk individuals are crucial for minimising the burden of cardiovascular diseases (CVDs). In this study, we aimed to evaluate the association of retinal microvascular features with myocardial dysfunction and CVD risk factors in a group of patients with significant coronary artery disease (CAD) compared to patients with newly diagnosed isolated arterial hypertension and healthy controls. *Materials and Methods*: We performed a single-centre cross-sectional study on 214 individuals divided into three groups: a group of 99 cases diagnosed with significant CAD, a group of 61 cases with newly diagnosed isolated arterial hypertension, and a control group of 54 cases with no confirmed cardiovascular pathology. Colour optic disc-centred retinal photographs were taken in all cases, and the following parameters were quantified using MONA REVA 3.0.0 software (VITO Health, Mol, Belgium): central retinal arteriolar equivalent, central retinal venular equivalent, arteriovenous ratio, fractal dimension, tortuosity index, and lacunarity. Univariable and multivariable statistical analyses were performed to assess changes in retinal microvascular features in CVD. *Results*: Dyslipidaemia (*p* = 0.009), systolic blood pressure (*p* = 0.008), and LDL cholesterol (*p* = 0.003) were negatively associated while left ventricular (LV) strain (0.043) was positively associated with the CRAE. In the case of the CRVE, the coronary Agatston score (*p* = 0.016) proved a positive and HDL cholesterol (*p* = 0.018) a negative association. A lower fractal dimension was associated with the presence of diabetes mellitus (*p* = 0.006), dyslipidaemia (*p* = 0.011), and a history of acute myocardial infarction (*p* = 0.018), while a higher fractal dimension was associated with increased left ventricular ejection fraction (LVEF) (*p* = 0.006) and medical treatment (*p* = 0.005). Lacunarity was higher in patients of female gender (*p* = 0.005), with decreased HDL (*p* = 0.014) and LVEF (0.005), and with increased age (*p* < 0.001) and Agatston score (*p* = 0.001). The vessel tortuosity index increased with LV strain (*p* = 0.05), medical treatment (*p* = 0.043), and male gender (*p* = 0.006). *Conclusions*: Retinal microvascular features may serve as additional risk stratification tools in patients with CVD, particularly CAD, pending prospective validation.

## 1. Introduction

Cardiovascular diseases (CVDs) remain a major cause of death worldwide, with an estimated 17.9 million cases each year (32% of all global deaths) (WHO). Even during the recent COVID-19 pandemic, ischemic heart disease was still the leading cause of death, with 113.7 deaths/100,000 population, surpassing the 109.7 deaths/100,000 population attributed to COVID-19 [1].

As life expectancy in industrialised countries continues to increase even further, according to projections [2], screening, primary prevention, and the early identification of high-risk individuals have become crucial for minimising the burden of cardiovascular diseases (CVDs). Pathophysiological events leading to CVD, like the development of atherosclerotic fatty streaks, begin as early as the teenage years, continuing into young adulthood [3]. Meanwhile, due to modern changes in lifestyle, severe atherosclerosis and related diseases are increasingly diagnosed in younger age groups [4,5].

Early detection and quantification of microvascular abnormalities, the substrate of both degenerative and atherosclerotic CVDs, could provide a valuable tool for screening and identifying individuals at risk for unfavourable outcomes.

Observable retinal vessels are arterioles and venules after the first or second bifurcation of the central retinal artery/vein [6] that provide an accessible view of the systemic microvascular circulation [7].

Shortly after the development of ophthalmoscopy in 1851, physicians all over the world began identifying retinal microvascular abnormalities in patients with CVD, especially in chronic arterial hypertension cases: arteriolar narrowing [8], increased arterial wall reflex, venous compression, and course distortion at arteriovenous crossings [9,10], tortuous arterioles, or venules with focal narrowing [11]. These changes were used for grading hypertensive retinopathy by Keith, Wagener, and Barker [12].

Modern digital fundus photography and optical coherence tomography angiography (OCTA) now allow the in vivo non-invasive quantitative and qualitative evaluation of retinal microcirculation.

In the case of digital fundus photography, formulas and algorithms have been developed and implemented into semi-automated computer programs to quantify arteriolar and venular diameters, arteriovenous ratios, vascular tortuosity, optimality, and fractal dimensions for the geometrical characterisation of arterial and venous components.

The prognostic significance of retinal vessel morphometric features has been investigated in CVDs such as arterial hypertension, diabetes, stroke, carotid artery stenosis, and coronary artery disease [13,14,15]. The observation of retinal arteriole narrowing and retinal venule lumen dilation has demonstrated additional predictive value for atherosclerotic CVD events in low-risk women, adding incremental value to the Pooled Cohort Equations used by the American College of Cardiology/American Heart Association to estimate the 10-year primary risk [16].

The current study aimed to evaluate the association of retinal microvascular features (vascular calibre and geometrical characteristics) with myocardial dysfunction and CVD risk factors in a group of patients with significant coronary artery disease (CAD) compared to patients with newly diagnosed isolated arterial hypertension and healthy controls.

## 2. Materials and Methods

A single-centre cross-sectional study was performed at the “Prof. Dr. George I.M. Georgescu” Cardiovascular Diseases Institute (Iasi, Romania) during the summer of 2024 on a population of 214 individuals addressed for diagnosis and treatment of CVD divided into three groups: a group of 99 cases diagnosed with significant chronic CAD requiring coronary artery bypass grafting or percutaneous transluminal coronary angioplasty (PTCA), a group of 61 cases with newly diagnosed isolated arterial hypertension (AHT), and a control group of 54 cases with no confirmed cardiovascular pathology.

Inclusion criteria for the study were as follows: age > 18 years and a good-quality fundus photograph of at least one eye. In the case of CAD patients, we enrolled 99 consecutive individuals with significant chronic CAD who were indicated for CABG or PTCA following coronary angiography (either conventional or computed tomography) in accordance with the 2018 ESC/EACTS Guidelines on Myocardial Revascularization [17]. In the case of the AHT group, we included patients with newly diagnosed isolated AHT, referred to our clinic by general practitioners. These individuals underwent a full evaluation, and no other CVD was detected apart from AHT. The control group consisted of individuals referred for routine assessments—such as pre-anaesthesia evaluation for non-vascular surgery, occupational cardiological screening, or interdisciplinary consultations—who had no diagnosed CVD. Patients with glaucoma, late-stage age-related macular degeneration, moderate or severe non-proliferative diabetic retinopathy, significant retinal vascular disease (central retinal artery or vein occlusion), proliferative diabetic retinopathy, significant refractive disorders (>4 dioptres), dense cataract, no gradable fundus photo, and cardiac arrhythmia impairing functional echocardiographic evaluation were excluded from the study.

This study was performed in accordance with the Declaration of Helsinki and approved by the medical ethics committee of the “Prof. Dr. George I.M. Georgescu” Cardiovascular Diseases Institute (Iasi, Romania) (approval number 2/1 July 2024). Written informed consent was obtained from every individual.

In all cases, we collected demographic data (age and gender), cardiovascular risk factors, cardiovascular and ophthalmological history, systemic medication, and laboratory test results. For patients diagnosed with CAD, coronary computed tomography angiography (CCTA) with calcium scoring and functional echocardiographic evaluation were performed, and two cardiovascular risk scores were calculated, PROCAM (Prospective Cardiovascular Munster) [18] and CHA2DS2-VASc [19]. For the functional echocardiographic evaluation, left ventricular (LV) global longitudinal strain values were determined from 2-, 3-, and 4-chamber view CINE images, and left atrial (LA) reservoir strain was determined from 4-chamber view CINE images acquired with a Philips Affiniti 50G ultrasound machine (Philips Healthcare, Best, Netherlands) and analysed using TomTec Arena version 2.51 software (TOMTEC Imaging Systems GmbH, Unterschleissheim, Germany).

**Figure 1:**
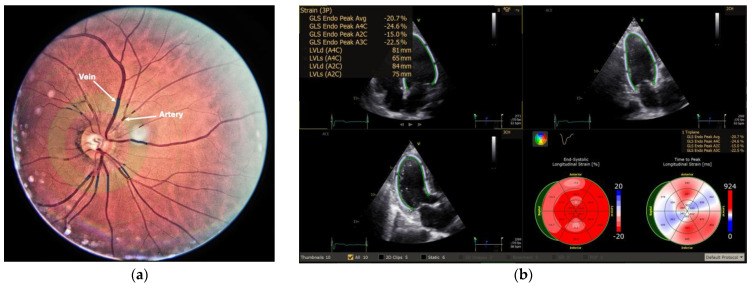
Retinal vessel evaluation with marked arteries, veins, and measurement zone (**a**) and left ventricular strain assessment (**b**).

Undilated 45° colour optic disc-centred retinal photographs were taken in all cases using a Nidek AFC-210 (NIDEK Co., Aichi, Japan) non-mydriatic auto-fundus camera after a 5 min dark adaptation period. After quality assessment, a single image (left or right eye) was chosen for each case. All images underwent pre-processing in ImageJ version 1.54 (National Institutes of Health, Bethesda, MD, USA) using automatic Contrast Limited Adaptive Histogram Equalization (CLAHE) for contrast enhancement. Pre-processed images were further analysed with MONA REVA version 3.0.0 software (VITO Health, Mol, Belgium; https://mona.health/) by two blinded independent graders [20]. The resolution was determined from the images in all cases, and the automatically detected optic disc contour was manually adjusted. The radius of the optic disc (OD) (900 μm) was used for image calibration (μm/pixel) and for delimiting three circles with 2, 3, and 5 times the radius of the OD. The vessels located in the zone between 2 and 3 times the radius of the OD were delineated automatically and manually edited to correct the accuracy of the analysis. The software then calculated the central retinal arteriolar equivalent (CRAE), central retinal venular equivalent (CRVE), and arteriovenous ratio (AVR) using the six largest arterioles and venules based on the modified formula by Knudtson et al. [21]. When anatomical variations did not allow the identification of 6 arterioles and 6 venules, a pair of 4 or 5 vessels was used. The vessel network between 1.5 and 5 times the radius of the OD was automatically segmented and skeletonised and then manually adjusted by deleting and/or retracing inaccurate segments. After adjusting, the geometry of the retinal vascular network was estimated using the fractal dimension (Df), lacunarity, and tortuosity index automatically quantified by the software. The fractal dimension measures geometric complexity, while lacunarity quantifies the inhomogeneity of vessel distribution based on the presence of gaps in a binary image. The tortuosity index is the average ratio of the end-to-end length to the actual length for all vessels in the marked area (Figure 1).

SPSS (version 26, IBM) was used for statistical analysis, and *p* values < 0.05 were considered significant.

Descriptive statistics were used to summarise baseline characteristics: mean ± standard deviation or median ± interquartile range and 1st and 3rd quartile for continuous variables depending on normality tests and counts/percentages for categorical variables. The Kolmogorov–Smirnov and Shapiro–Wilk tests were used to assess normality.

The CRAE, CRVE, Df, lacunarity, and tortuosity index were analysed as both continuous and categorical variables grouped according to quartiles. Systolic blood pressure (SBP) < 140 mmHg and diastolic blood pressure (DBP) < 90 mmHg were considered normal.

An independent samples T-Test or the Mann–Whitney U test was used to compare two groups, while a one-way ANOVA or the Kruskal–Wallis test was used to compare more independent samples according to tests of normality results.

The Chi-square test was used to evaluate the association between categorical variables, Pearson correlation was used for the association between continuous variables, and univariable linear regression was used to assess the association of categorical variables with continuous variables.

In multiple regression, we first included associated factors (*p* < 0.1) from the initial models, and in a second step, we performed an exploratory stepwise multiple regression with a backward selection procedure. To give a more comprehensive picture of the effect of independent variables on the dependent variable, we performed quantile regression.

## 3. Results

The descriptive statistics for all baseline characteristics and a comparison of significance between groups are detailed in Table 1. All continuous baseline characteristics proved a normal distribution when tested with Kolmogorov–Smirnov and Shapiro–Wilk tests. As anticipated, the three groups differed significantly in terms of age, gender distribution, SBP, total cholesterol, HDL and LDL cholesterol, triglycerides, the prevalence of diabetes mellitus, and acute myocardial infarction: CAD and hypertensive patients were older and mostly males, diabetes registered a higher prevalence in the CAD group, higher SBP was registered in the hypertensive group, and higher total, HDL, and LDL cholesterol and serum triglycerides were registered in hypertensive and control groups. It should be considered that the differences in lipid profiles, especially the low LDL levels in the CAD group, may be largely related to the effect of statins or similar treatments.

Retinal microvascular characteristics of the studied population and comparison results are summarised in Table 2. Lacunarity and the tortuosity index did not prove a normal distribution and were presented and tested accordingly. Both the CRAE and CRVE registered lower values in the CAD group compared to controls and AHT, while the fractal dimension (Df) was lower in patients with arterial hypertension. Lacunarity did not register significant differences between groups. The retinal vessel tortuosity index was lower in CAD patients compared to the control and AHT groups.

Retinal microvascular parameters registered a significant association with the presence of cardiovascular diseases (AHT and CAD) when tested with linear regression, both unadjusted and adjusted for medical treatment (shown in Table 3).

In patients with confirmed significant CAD, we tested the association of retinal microvascular parameters with known risk factors, diagnosis, and medical treatment (number of administered drugs). Table 4 summarises the Pearson correlation coefficient between retinal microvascular parameters and the continuous variables included in the analysis.

After univariable analysis, age proved a negative correlation with fractal dimensions (*p* = 0.007) and a positive correlation with lacunarity (*p* = 0.049) that were confirmed by the multiple regression. HDL cholesterol showed a negative correlation with the CRVE (*p* = 0.049), also confirmed by the multiple regression. SBP showed a negative correlation with both the CRAE (*p* = 0.040) and AVR (*p* = 0.034), both confirmed by the multiple regression. The PROCAM score showed a positive correlation with the CRVE (*p* = 0.016) but was unconfirmed by the multiple regression, and the AGATSTON score showed a positive correlation with the CRVE (*p* = 0.008), also confirmed by the multiple regression.

Table 5 summarises the univariable linear regression results for the retinal microvascular parameters and categorical variables.

The univariable linear regression identified gender as a potential predictor for the CRAE and AVR, although it was not confirmed by the multiple regression analysis, while dyslipidaemia was identified and confirmed as a potential predictor for CRAE (*p* = 0.005) by multiple regression.

After the univariable analysis, an exploratory stepwise multiple regression with a backward selection procedure was performed to identify the best models predicting retinal microvascular parameters variance to create the framework for a personalised risk assessment and therapeutic management potentially based on retinal microvascular parameters.

The results displayed in Table 6 show that dyslipidaemia, SBP, LV strain, and LDL cholesterol are statistically significantly associated with the CRAE in the multivariable linear regression analysis. The R square value of 0.427 indicates that 42.7% of the total CRAE variance could be attributed to independent factors in the regression model (*p* = 0.005). More specifically, SBP over 140 mmHg, the presence of dyslipidaemia, and increased LDL cholesterol were associated with decreased CRAE values in our group of CAD patients. On the other hand, increased LV strain was associated with increased CRAE values.

In the case of the CRVE, only the coronary Agatston score and HDL cholesterol proved a statistically significant association, with an R squared value of 0.713, thus indicating that 71.3% of the CRVE variance could be explained by the regression model (*p* = 0.004). A higher coronary Agatston score and lower HDL were associated with an increased CRVE.

For the AVR, the regression model explained only 8.5% of the total variance (R square 0.085, *p* = 0.018), with a weak positive association between higher SBP and triglyceride levels with an increased AVR.

A lower fractal dimension was associated with the presence of diabetes mellitus, dyslipidaemia, and a history of acute myocardial infarction, while a higher fractal dimension was associated with an increased LVEF and medical treatment. The regression model explained 44.4% of total variance (R square = 0.444, *p* = 0.012).

Lacunarity, a measure of the inhomogeneity of the retinal microvascular tree, was higher in patients of female gender, with decreased HDL and LVEF and with increased age and Agatston score. The regression model explained 95.2% of the total variance (R square = 0.952, *p* = 0.004).

The vessel tortuosity index increased with LV strain, medical treatment, and male gender, with the regression model explaining only 28.1% of the total variance (R square 0.281, *p* = 0.008).

As the effect of independent predictors could vary, we performed a quantile regression to estimate an independent variable’s predictive effect on a specified quantile of our dependent variables (Table 7). In the case of the CRAE, the effect of the regression model was higher for the median and upper quantiles, while for the CRVE it was similar for all quantiles. For the AVR, the prediction value was higher in the upper quantile but with increased mean absolute error, while for fractal dimensions, it was higher in the lower quantile. On the opposite, for lacunarity, the predictive value was higher for the lower and upper quantiles, while for the tortuosity index, it was higher in the lower quantile.

## 4. Discussion

The present research proved an association between retinal microvascular parameters and CVD, such as newly diagnosed AHT and significant CAD requiring CABG or PTCA. The CRAE, CRVE, Df, and tortuosity index were significantly associated with the presence of both AHT and CAD before and after adjusting for medical treatment.

In particular, the CRAE, CRVE, and retinal vessel tortuosity index registered lower values in the CAD group compared to controls and AHT, potentially because of longstanding atherosclerosis and a more severe CVD profile involving systemic endothelial dysfunction and microvascular inflammation. On the other hand, the fractal dimension was significantly lower in patients with newly diagnosed AHT compared to CAD and controls. This could be attributed to the higher blood pressure values in patients with newly diagnosed AHT (mean SBP 155.71 ± 18.06 mmHg) compared to CAD patients (147.02 ± 19.04 mmHg).

In larger studies, like those performed by McGowan et al. [22] on 1122 participants and Dervenis et al. [23] on 1614 participants, AHT was also significantly associated with a narrower CRAE. Dervenis et al. additionally proved that BP normalisation under antihypertensive treatment reversed the retinal microvascular profile and that higher DBP was associated with a decreased value of CRVE in patients aged 80 years or older.

An extensive meta-analysis on the relationship between retinal vessel calibre with CAD and CAD risk factors was performed by Liang et al. [24] on 14 studies published from 2001 to 2021. According to this meta-analysis, a lower CRAE was related to CAD, and no significant difference in the CRVE between CAD and healthy controls was found, in contrast to our study that identified lower CRVE values (236.33 ± 28.89 µm) in significant CAD patients compared to healthy controls (251.66 ± 20.72 µm). Retinal vascular calibre is also independently related to CAD risk factors like age, gender, smoking, BMI, AHT, and diabetes mellitus, the same as in our study.

The most extensive investigations on the association between cardiometabolic risk factors and retinal microvascular parameters in the general population were performed by Owen et al. [25] on a group of 5947 participants enrolled in the European Prospective Investigation into Cancer (EPIC) study and by Chandra et al. [13] on a group of 10692 individuals enrolled in the Atherosclerosis Risk in Communities (ARIC) study. These studies concluded that narrower arterioles were associated with age and higher SBP and total and HDL cholesterol levels even after excluding patients with AHT and CVD. Increased arteriolar tortuosity was associated with age, higher SBP, and patients with prevalent stroke. Increased venular tortuosity was associated with higher BMI, haemoglobin A1c (HbA1c) level, and type 2 diabetes mellitus. Wider venules were associated with older age, higher triglyceride levels, BMI, HbA1c level, and smoking. Atherosclerotic risk factors generally correlated more with the CRAE and arteriolar tortuosity. The CRVE increase and CRAE decrease were associated with larger LV size, a higher prevalence of LV hypertrophy, and decreased diastolic and systolic function.

French et al. [26] attempted to stratify cardiovascular risk in the general population according to AVR cut-off values and concluded that CVD risk was significantly higher in patients with AVR in the lowest quintile compared to the highest (QRISK: 14.28% vs. 9.87% [*p* = 0.05]; MAYO risk: 36.35% vs. 19.21% [*p* = 0.01]).

In the particular case of patients with significant CAD, age, HDL cholesterol, SBP, and cardiovascular risk scores (PROCAM and Agatston) had a significant impact on retinal microvascular parameters after the univariable analysis. Fractal dimensions decreased with age while lacunarity increased; the CRVE increased with decreasing HDL cholesterol, while the CRAE decreased with increasing SBP.

In the multivariable analysis, dyslipidaemia, SBP, LV strain, and LDL cholesterol explained 42.7% of the total CRAE variance. Our study is the first to investigate the association of cardiac functional parameters (LV and LA strain) with retinal microvascular factors. Increased left ventricular contractility quantified by LV strain correlated with higher CRAE values. To date, no published studies have analysed the association between retinal microvascular features and LV strain. However, several studies have demonstrated that resting global strain is predictive of exercise ability in the treadmill stress test [27,28]. Thus, in CAD patients, CRAE values in the upper percentile could be an indicator of good functional reserve and potentially increased exercise tolerance and rapid recovery of myocardial function after coronary revascularisation. On the other hand, lower CRAE values are associated with a high cardiovascular risk and disease severity. In the study performed by Seecheran et al. [29], retinal arterial diameters were negatively correlated with the SYNTAX angiographic score, an important prognostic tool in patients undergoing percutaneous coronary intervention. Wang et al. [30] reached similar results when evaluating CAD severity using the Gensini score: the CRAE and AVR were reduced to a greater extent in patients with severe and moderate CAD compared to patients with mild CAD.

On the other hand, the coronary Agatston score and HDL cholesterol explained 71.3% of CRVE variance. Compared to the CRAE, CRVE values in the upper percentile could be an indicator of severe and diffusely calcified coronary atherosclerotic plaques based on the Agatston score.

The limited association between lipid profiles and retinal microvascular features may be influenced by lipid-lowering therapy in CAD patients. Since these patients had severe atherosclerotic lesions, they were already on lipid-lowering medications (primarily statins) at the time of retinal imaging. The complexity of the retinal microvascular network based on fractal dimensions was increased in patients with higher LVEF values and thus a better prognosis, while a more severe comorbidity status (diabetes mellitus, dyslipidaemia, and history of acute myocardial infarction) was associated with a lower retinal microvascular network complexity in our study. This was confirmed by another parameter, lacunarity, whose increased values were associated with a lower LVEF.

Increased LV contractility (systolic function) was also associated with an increased tortuosity index, with straighter vessels being a marker of decreased myocardial functional reserve. Compared to Wei et al. [31], we identified no association of retinal microvascular parameters with diastolic LV dysfunction, with mention that LA strain was included only as an indicator of diastolic dysfunction in the current study. Wei at al. showed that lower retinal microvascular fractal dimensions were also associated with an increased E/e′ ratio, reflecting an increase in the LV filling pressure.

Wang et al. [30] investigated the joint effect of multiple retinal vessel parameters on CAD and showed that straighter retinal vessels and a lower Df were associated with CAD extent and Gensini angiographic scores, factors with a significant impact on LV function. Fu et al. [32] reached similar results in a study on 57947 participants enrolled in UK Biobanks and followed for 11 years. In their study, decreased complexity and density of the retinal microvascular network indicated an increased risk of incident CAD. Another study performed by Sandoval-Garcia et al. [33] on a group of 1066 individuals diagnosed with type 2 diabetes mellitus, out of which 200 developed an incident cardiovascular event (CAD or cerebrovascular) over a follow-up period of 8 years, identified a significant association of the Df with incident cerebrovascular events (HR 0.73 [95% CI 0.57, 0.95]) and stroke (HR 0.72 [95% CI 0.54, 0.97]) and a weak, non-significant association with CAD.

In the research performed by Dinesen et al. [34], CAD patients with a Df in the fourth quartile were less likely to have CABG compared to patients in the first quartile (OR, 7.20; 95% CI: 1.63–31.86; *p* = 0.009).

Our results and the literature analysis prove that retinal microcirculation correlates well with coronary macro- and microcirculation alterations. Several aspects should be analysed and corroborated when investigating retinal microcirculation: vascular geometrical complexity (revealed by Df and lacunarity), general morphology (quantified as tortuosity), and vessel calibre (CRAE, CRVE, and AVR), as they reflect both structural and functional cardiovascular changes.

Future research on large groups of CAD patients with various disease severity and including functional myocardial assessment is necessary to better understand the prognostic value of retinal microvascular parameters to develop risk and prognostic models.

This study has several methodological and analytical limitations. Due to its cross-sectional design and relatively small sample size, future prospective studies with larger cohorts and stricter selection criteria are required to corroborate the findings. Additionally, the heterogeneity of the study population and the inability to control for potential specific drug-related effects (the majority of CAD patients received active medical treatment at the time of retinal imaging) may have influenced the assessment of retinal microvascular parameters.

## 5. Conclusions

Retinal microvascular parameters such as the central retinal arteriolar equivalent, central retinal venular equivalent, fractal dimension, lacunarity, and tortuosity correlate with cardiovascular risk factors and disease severity in patients with coronary artery disease and could prove to be potentially useful tools for both screening and risk stratification as part of novel risk stratification scores pending prospective validation.

## Figures and Tables

**Table 1:** Baseline characteristics of the studied population.

Variable	Control	AHT	CAD	*p*
Age (years) (mean ± SD)	58.9 ± 9.15	60.43 ± 12.40	64.08 ± 10.90	0.0121
Male gender (no., %)	29 (53.70%)	37 (60.66%)	76 (76.77%)	0.0083
Current smoker (no., %)	5 (9.26%)	5 (8.20%)	4 (4.04%)	n.s.
Diabetes mellitus (no., %)	2 (3.70%)	10 (16.39%)	41 (41.41%)	<0.001
Mild diabetic retinopathy (no., %)	0%	0%	3 (3.03%)	-
AMI (no., %)	0%	0%	2 (2.02%)	-
Stroke (no., %)	0%	3 (4.92%)	4 (4.04%)	-
CKD (no., %)	2 (3.70%)	6 (6.59%)	8 (8.08%)	n.s.
SBP (mmHg) (mean ± SD)	127.52 ± 10.52	155.71 ± 18.06	147.02 ± 19.04	<0.001
Total cholesterol (mg/dL) (mean ± SD)	189.5 ± 27.18	195 ± 32.57	162.76 ± 52.22	<0.001
HDL cholesterol (mg/dL) (mean ± SD)	54.1 ± 10.51	45.38 ± 14.74	43.07 ± 10.89	<0.001
LDL cholesterol (mg/dL) (mean ± SD)	139.2 ± 28.63	116.85 ± 28.82	97.88 ± 43.23	<0.001
TG (mg/dL) (mean ± SD)	177.1 ± 106.3	172.15 ± 96.05	141.64 ± 78.59	0.0324

AHT: arterial hypertension; CAD: coronary artery disease; AMI: acute myocardial infarction; CKD: chronic kidney disease; SBP: systolic blood pressure; HDL: high-density lipoprotein; LDL: low-density lipoprotein; TG: triglycerides; SD: standard deviation; n.s.: non-significant.

**Table 2:** Retinal microvascular characteristics of the studied population.

Variable	Control	AHT	CAD	*p*
CRAE (µm, mean ± SD)	172.79 ± 14.32	165.75 ± 17.67	157.59 ± 16.75	<0.001
CRVE (µm, mean ± SD)	251.66 ± 20.72	253.53 ± 33.87	236.33 ± 28.89	<0.001
AVR (mean ± SD)	0.69 ± 0.058	0.66 ± 0.082	0.67 ± 0.078	n.s.
Fractal dimension (mean ± SD)	1.43 ± 0.030	1.40 ± 0.043	1.42 ± 0.044	<0.001
Lacunarity (median ± IQR; 25–75%)	1.063 ± 0.035 (1.044–1.079)	1.065 ± 0.044 (1.043–1.087)	1.073 ± 0.034 (1.054–1.088)	n.s.
Tortuosity index (median ± IQR; 25–75%)	0.884 ± 0.011 (0.879–0.890)	0.880 ± 0.019 (0.871–0.890)	0.876 ± 0.025 (0.865–0.890)	0.037

IQR: interquartile range; SD: standard deviation; n.s.: non-significant.

**Table 3:** Association of retinal microvascular parameters with cardiovascular diseases.

Variable		Beta	Standard Error	Adjusted R^2^	*p*
CRAE	Single	−0.349	0.734	0.118	<0.001
	+medical treatment	−0.100	0.003	0.641	0.023
CRVE	Single	−0.205	0.003	0.038	0.003
	+medical treatment	0.015	0.002	0.633	0.004
Df	Single	−0.041	1.955	−0.003	0.547
	+medical treatment	−0.150	1.157	0.655	<0.001
Tortuosity index	Single	−0.191	4.800	0.032	0.005
	+medical treatment	−0.060	2.985	0.636	0.153

CRAE: central retinal arteriolar equivalent; CRVE: central retinal venular equivalent; Df: fractal dimension.

**Table 4:** Pearson correlation coefficient for retinal microvascular parameters and continuous variables.

Variable		CRAE	CRVE	AVR	Df	Lacunarity	Tortuosity Index
Age	Correlation coefficient	0.034	−0.07	0.117	−0.271	0.198	0.010
	*p* value	0.736	0.491	0.249	0.007	0.049	0.922
LDL	Correlation coefficient	−0.256	−0.15	−0.072	−0.068	−0.078	0.054
	*p* value	0.022	0.185	0.524	0.548	0.492	0.636
HDL	Correlation coefficient	−0.167	−0.220	0.076	0.021	0.067	−0.070
	*p* value	0.139	0.049	0.502	0.853	0.556	0.538
Triglycerides	Correlation coefficient	0.007	0.123	−0.125	0.058	0.044	−0.069
	*p* value	0.946	0.225	0.216	0.568	0.668	0.498
LVEF	Correlation coefficient	0.022	−0.033	0.066	−0.102	−0.087	0.097
	*p* value	0.826	0.750	0.518	0.319	0.395	0.343
SBP	Correlation coefficient	−0.028	−0.191	−0.220	0.053	0.005	−0.141
	*p* value	0.040	0.065	0.034	0.615	0.964	0.176
DBP	Correlation coefficient	−0.014	−0.040	0.009	0.050	0.035	−0.106
	*p* value	0.891	0.704	0.928	0.632	0.739	0.308
LV strain	Correlation coefficient	0.030	0.058	−0.038	−0.137	0.049	0.256
	*p* value	0.857	0.727	0.817	0.407	0.765	0.116
LA strain	Correlation coefficient	0.077	−0.133	0.231	−0.052	−0.182	−0.095
	*p* value	0.669	0.462	0.195	0.773	0.311	0.598
CHA_2_DS_2_-VASc Score	Correlation coefficient	0.090	0.038	0.028	−0.140	−0.169	−0.0933
	*p* value	0.376	0.711	0.783	0.168	0.094	0.361
PROCAM score	Correlation coefficient	0.245	0.404	−0.258	0.115	0.100	−0.056
	*p* value	0.156	0.016	0.135	0.511	0.569	0.750
Agatston score	Correlation coefficient	0.186	0.676	−0.359	−0.222	−0.046	0.446
	*p* value	0.523	0.008	0.208	0.445	0.876	0.110

LVEF: left ventricular ejection fraction; LV: left ventricle; LA: left atrium; DBP: diastolic blood pressure; SBP: systolic blood pressure.

**Table 5:** Univariate linear regression for retinal microvascular parameters and categorical variables.

Variable	CRAE		CRVE		AVR		Df		Lacunarity		Tortuosity Index	
	*p* Value	B	*p* Value	B	*p* Value	B	*p* Value	B	*p* Value	B	*p* Value	B
Gender	0.030	−1.520	0.592	−3.711	0.069	−0.001	0.941	−0.001	0.981	0.005	0.307	0.005
Dyslipidaemia	0.005	−6.770	0.101	−11.674	0.812	−0.005	0.842	−0.002	0.489	−0.006	0.350	0.004
Current smoker	0.317	0.102	0.425	−0.081	0.097	0.168	0.404	0.085	0.335	0.098	0.691	0.040
Diabetes mellitus	0.659	0.777	0.947	−0.204	0.999	1.527	0.962	0.005	0.734	0.001	0.199	−0.003
Prior stroke	0.680	3.553	0.398	12.553	0.544	−0.025	0.811	0.005	0.285	−0.019	0.786	0.003
Prior AMI	0.818	0.799	0.124	9.046	0.233	−0.020	0.586	0.005	0.427	0.006	0.308	−0.004
NYHA class	0.733	−0.035	0.985	−0.002	0.714	−0.037	0.544	0.062	0.613	0.052	0.827	−0.022
Therapeutic intervention	0.259	−0.005	0.307	−0.003	0.217	−0.002	0.312	1.617	0.354	1.896	0.199	−4.850

CRAE: central retinal arteriolar equivalent; CRVE: central retinal venular equivalent; AVR: arteriovenous ratio; Df: fractal dimension; AMI: acute myocardial infarction; NYHA: New York Heart Association.

**Table 6:** Multivariate linear regression models.

Model	Unstandardised Coefficients		Standardised Coefficients	t	*p*
	B	Std. Error	Beta		
***CRAE***					
(Constant)	221.977	20.832		10.655	0.000
Dyslipidaemia	−15.926	5.603	−0.430	−2.842	0.009
SBP	−17.091	5.983	−0.493	−2.856	0.008
LV strain	1.784	0.954	0.323	1.870	0.043
LDL	−0.195	0.060	−0.510	−3.242	0.003
***CRVE***					
(Constant)	278.330	25.342		10.983	0.000
Agatston Score	0.031	0.011	0.541	2.940	0.016
HDL	−1.486	0.515	−0.531	−2.888	0.018
***AVR***					
(Constant)	0.542	0.063		8.639	0.000
SBP	−0.001	0.000	−0.258	−2.524	0.013
TG	0.000	0.000	−0.195	−1.907	0.060
***Df***					
(Constant)	1.704	0.083		20.409	0.000
LVEF	0.004	0.001	0.629	3.014	0.006
DM	−0.021	0.008	−0.431	−2.650	0.014
AMI	−0.052	0.021	−0.537	−2.515	0.018
Medical treatment	0.035	0.011	0.508	3.088	0.005
Dyslipidaemia	−0.050	0.018	−0.465	−2.752	0.011
Age	−0.033	0.022	−0.223	−1.452	0.158
***Lacunarity***					
(Constant)	1.376	0.041		33.874	0.000
HDL	−0.001	0.000	−0.569	−3.693	0.014
Age	0.002	0.000	1.056	8.600	0.000
LVEF	−0.002	0.000	−0.484	−4.734	0.005
Agatston Score	3.664	0.000	0.841	6.841	0.001
Gender	−0.046	0.010	−0.740	−4.812	0.005
***Tortuosity index***					
(Constant)	0.905	0.020		45.227	0.000
Gender	0.022	0.008	0.431	2.914	0.006
LV strain	0.002	0.001	0.292	2.026	0.050
Medical treatment	0.010	0.005	0.312	2.101	0.043

CRAE: central retinal arteriolar equivalent; CRVE: central retinal venular equivalent; AVR: arteriovenous ratio; Df: fractal dimension; AMI: acute myocardial infarction; DBP: diastolic blood pressure; SBP: systolic blood pressure; DM: diabetes mellitus; LV: left ventricle; LA: left atrium; LVEF: left ventricular ejection fraction; HDL: high-density lipoprotein; LDL: low-density lipoprotein. TG: triglycerides.

**Table 7:** Model quality assessment with quantile regression.

	q = 0.25	q = 0.5	q = 0.75
***CRAE***			
Pseudo R Squared	0.179	0.359	0.413
MAE	11.0223	8.7129	9.8379
***CRVE***			
Pseudo R Squared	0.536	0.515	0.554
MAE	14.1492	12.4089	16.9146
***AVR***			
Pseudo R Squared	0.363	0.261	0.418
MAE	0.0691	0.0631	0.0984
***Df***			
Pseudo R Squared	0.359	0.303	0.324
MAE	0.0266	0.0246	0.0357
***Lacunarity***			
Pseudo R Squared	0.376	0.269	0.575
MAE	0.0287	0.0254	0.0286
***Tortuosity index***			
Pseudo R Squared	0.212	0.195	0.147
MAE	0.0208	0.0140	0.0192

CRAE: central retinal arteriolar equivalent; CRVE: central retinal venular equivalent; AVR: arteriovenous ratio; Df: fractal dimension; MAE: mean absolute error.

## Data Availability

Data supporting the findings of this study are not publicly available due to confidential information but are available from the corresponding authors upon reasonable request.
